# High detection rate in [^18^F]PSMA-1007 PET: interim results focusing on biochemical recurrence in prostate cancer patients

**DOI:** 10.1007/s12149-021-01602-x

**Published:** 2021-03-04

**Authors:** Tadashi Watabe, Motohide Uemura, Fumihiko Soeda, Sadahiro Naka, Takeshi Ujike, Koji Hatano, Hidetaka Sasaki, Takashi Kamiya, Eku Shimosegawa, Hiroki Kato, Jens Cardinale, Ukihide Tateishi, Norio Nonomura, Frederik L. Giesel

**Affiliations:** 1 Department of Nuclear Medicine and Tracer Kinetics, Graduate School of Medicine, Osaka University, 2-2 Yamadaoka, Suita, Osaka 565-0871 Japan; 2 Institute for Radiation Sciences, Osaka University, Osaka, Japan; 3 Department of Urology, Graduate School of Medicine, Osaka University, Osaka, Japan; 4 Department of Radiology, Osaka University Hospital, Osaka, Japan; 5 Department of Molecular Imaging in Medicine, Graduate School of Medicine, Osaka University, 2-2 Yamadaoka, Suita, Osaka 565-0871 Japan; 6 Department of Nuclear Medicine, University Hospital Heidelberg, Heidelberg, Germany; 7 Department of Diagnostic Radiology, Medical Hospital, Tokyo Medical and Dental University, Tokyo, Japan; 8 Department of Nuclear Medicine, University Hospital Düsseldorf, Düsseldorf, Germany

**Keywords:** PSMA, PET, Prostate cancer, Biochemical recurrence, Diagnosis

## Abstract

**Objective:**

^18^F-labeled prostate-specific membrane antigen (PSMA) ligand, [^18^F]PSMA-1007, has the benefit of a higher synthetic yield and minimal excretion in the urine. High detection efficacy was reported in biochemical recurrence (BCR) of prostate cancer after radical prostatectomy. Thus, we evaluated the preliminary diagnostic utility of [^18^F]PSMA-1007 PET in patients with prostate cancer, focusing on the BCR which is not detected on conventional imaging.

**Methods:**

We enrolled a total of 28 patients (age 51–79 years) with BCR of prostate cancer. BCR was defined as a continuous increase in PSA after radical prostatectomy or radiation therapy without any apparent recurrent lesions on conventional diagnostic imaging (CT and bone scintigraphy). PSMA-PET scanning was performed approximately 60 min after intravenous injection of [^18^F]PSMA-1007 (259 ± 37 MBq). PSMA-PET images were evaluated for lesion detection as well as its relation to PSA values and location.

**Results:**

Abnormal uptake, which was suspected to be recurrence or metastasis, was detected in 92.9% (26/28) of patients with BCR. The SUVmax was 8.4 ± 6.4 in local recurrence, 11.5 ± 11.8 in pelvic lymph nodes (LN), and 4.1 ± 1.6 in bone metastasis. The detection rates were 66.7% in the PSA group-1 (0.1–0.5 ng/mL), 85.7% in the PSA group-2 (0.5–1.0 ng/mL), and 100% in the PSA group-3 (above 1.0 ng/mL). Among the PET-positive BCR patients (*n* = 26), local recurrence was detected in 57.7% (15/26), pelvic LN in 42.3% (11/26), and bone metastasis in 15.4% (4/26). In 53% (8/15) of BCR patients who were suspected of local recurrence, focal uptake was detected adjacent to the bladder on [^18^F]PSMA-1007 PET. This suggested the significant advantage of having minimal physiological urine excretion.

**Conclusions:**

[^18^F]PSMA-1007 PET showed a high detection rate in recurrent and metastatic lesions. In patients with BCR, its high detection led to suitable treatment strategies, such as salvage radiation therapy or surgical removal of recurrent lymph nodes.

**Trial registration:**

(UMIN Clinical Trials Registry) UMIN000037697.

## Introduction

Prostate-specific membrane antigen (PSMA) is a cell membrane-bound protein that is highly expressed in prostate cancer cells and the neovasculature of other tumors [1]. Expression of PSMA was observed in most prostate cancer patients and was positively correlated with Gleason score, a histological marker for degree of malignancy [2, 3]. PSMA expression is preserved or increased after androgen deprivation therapy (ADT) in patients with metastatic CRPC (castration-resistant prostate cancer) [4]. Thus, PSMA-PET can be used for a wide variety of purposes in prostate cancer patients, from initial staging and detection of recurrence, to pre-treatment evaluation of PSMA-targeted radionuclide therapy using [^177^Lu]Lu-/[^225^Ac]Ac-PSMA-617.

[^68^ Ga]Ga-PSMA-11 PET has been mainly used for PET imaging targeting PSMA and was recently approved by the FDA as the 1st PSMA PET probe. Meanwhile, the ^18^F-labeled PSMA ligand, [^18^F]PSMA-1007, has the advantages of the abundant availability of ^18^F and higher synthetic yield. It also showed favorable biodistribution in humans, with minimal excretion in the urine [5]. High detection efficacy has been reported in biochemical recurrence (BCR) of prostate cancer after radical prostatectomy using [^18^F]PSMA-1007 PET [6]. Its diagnostic accuracy was also confirmed for lymph node staging of prostate carcinoma in primary and biochemical recurrence compared to histological findings [7]. In this study, we evaluated the preliminary diagnostic utility of [^18^F]PSMA-1007 PET in patients with prostate cancer, focusing on the BCR, as an interim report.

## Materials and methods

### The study protocol and patient population

A total of 28 patients (age 51–79 years) with prostate cancer were included in this prospective study. Inclusion criteria were patients diagnosed with prostate cancer and who underwent CT and bone scintigraphy (BS) and showed continuously increased PSA after local treatment with radical prostatectomy or radiation therapy, with no apparent recurrent lesion on CT and BS. Patient characteristics are summarized in Table 1. The study protocol was approved by the institutional review board of Osaka University Hospital and the study has, therefore, been performed in accordance with the ethical standards laid down in the 1964 Declaration of Helsinki. Written informed consent was obtained from all patients prior to their inclusion in the study.

**Table 1:** Clinical characteristics of the patients

Characteristics	
Age at PET [median (range)]	67.5 (51–79) year
PSA at PET [median (range)]	2.39 (0.12–39.78) ng/mL
Gleason score	
6	5
7	12
8	4
9	7

### Imaging protocol

[^18^F]PSMA-1007 solution was synthesized using MPS200 (Sumitomo Heavy Industries) according to a previous study [8, 9]. After at least 2 h of fasting, PSMA-PET scanning was performed approximately 60 min (57.7 ± 4.9 min, range 47.5–65.5) after intravenous injection of [^18^F]PSMA-1007 (259 ± 37 MBq). PET/CT images were acquired using Discovery 710 (GE, Milwaukee, United States) in 3-D mode (matrix 192 × 192, pixel size 3.65 mm) with 2 min per bed position. PET images were reconstructed using an ordered subset expectation maximization (OSEM) algorithm with three iterations per eight subsets, and Gauss-filtered to a transaxial resolution of 4 mm at full-width at half-maximum (FWHM). Attenuation correction was performed using the unenhanced low-dose CT data (tube current 100 mA). The CT-scans were reconstructed to a slice thickness of 3.75 mm with an increment of 3.27 mm. CT and BS were performed according to the institutional standard protocol as routine clinical practice.

### Data analysis

PSMA-PET images were analyzed for detection of lesions, as well as their relation to PSA values and location in patients with BCR. All PET images were interpreted by two physicians with board certifications. When there were differences in the interpretation of a result, it was judged by the senior nuclear medicine physician who has enough knowledge about PSMA-PET imaging. Detection rate was defined as the image-based positive finding which was suspected recurrence on PSMA-PET. True positivity was defined based on the histopathological confirmation (*n* = 5) or clinical course [PSA decline after the radiation therapy to PSMA-positive lesions (*n* = 11), decreased uptake on PSMA-PET after systemic therapy along with PSA decline or increase in size on CT (*n* = 4)]. In other cases without sufficient evidence (*n* = 6), apparent abnormal uptakes were judged as positive in potentially recurrent or metastatic regions. The impact on patient management was also evaluated after PSMA-PET in patients with BCR.

## Results

**Fig. 1:**
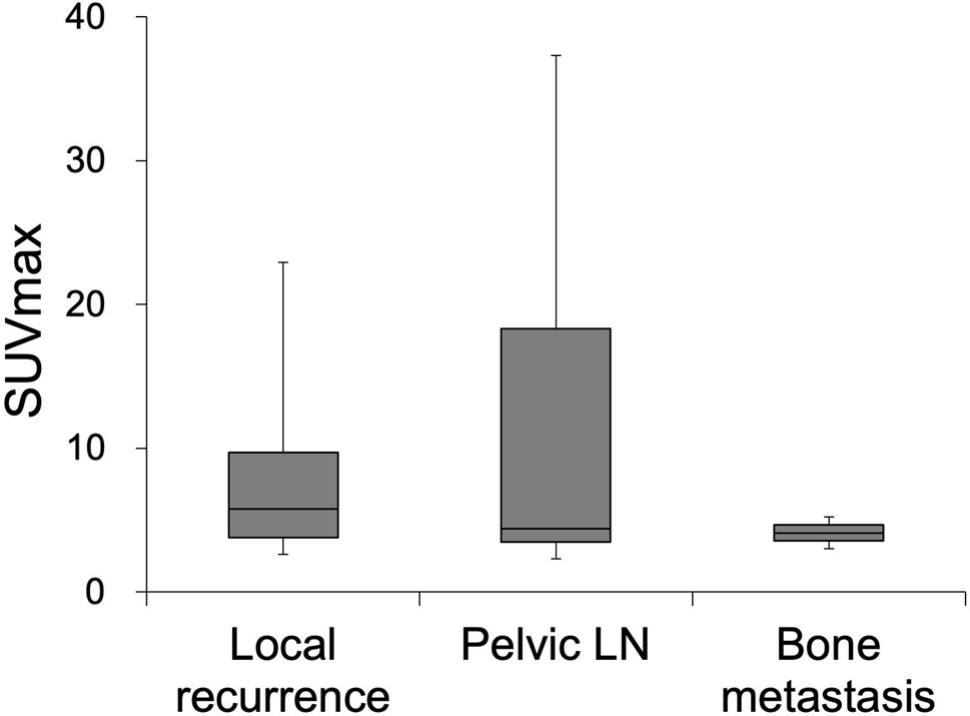
Uptake in recurrent lesions on [^18^F]PSMA-1007 PET (Pelvic LN: pelvic lymph nodes)

**Fig. 2:**
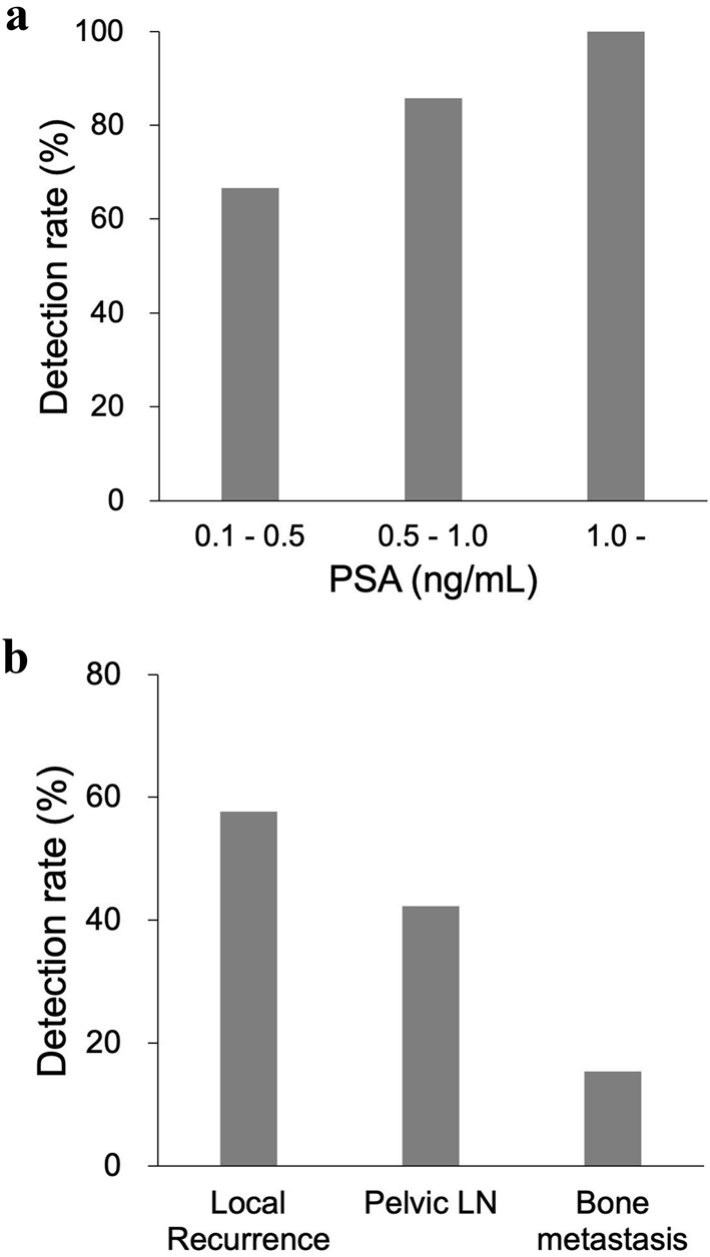
**a** Detection rates and their relation to PSA values and **b** location in BCR patients

**Fig. 3:**
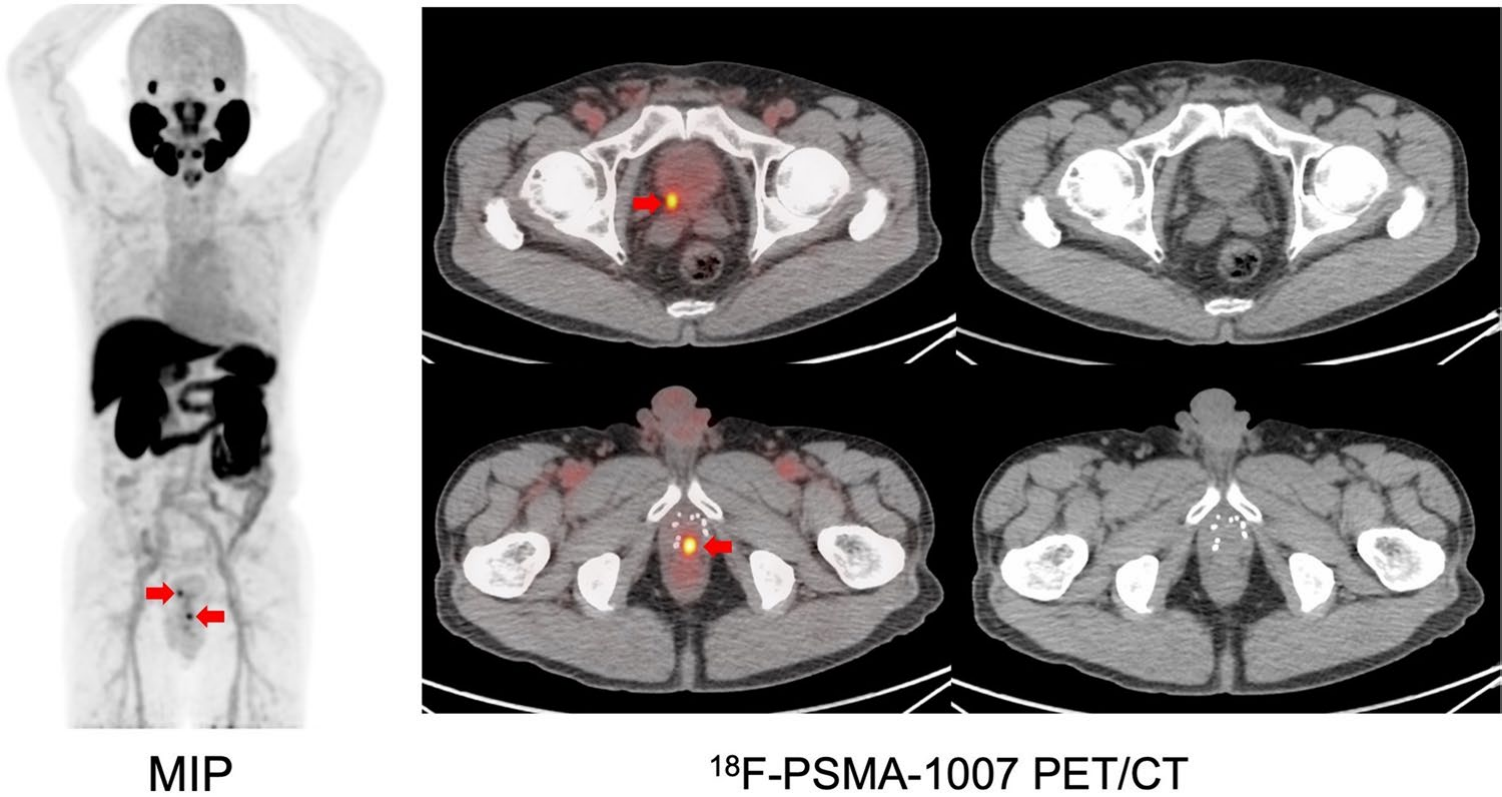
[^18^F]PSMA-1007 PET in BCR patient after ^125^I-seed implantation (PSA at PET: 3.32 ng/mL) (MIP: maximum intensity projection). Focal uptakes are observed on PSMA-PET (red arrows) and biopsy on the caudal lesion revealed a recurrence. Radiation therapy (cyber-knife) is performed targeting the two lesions and PSA value shows a decrease (PSA at 7 months after cyber-knife: 0.61 ng/mL)

**Fig. 4:**
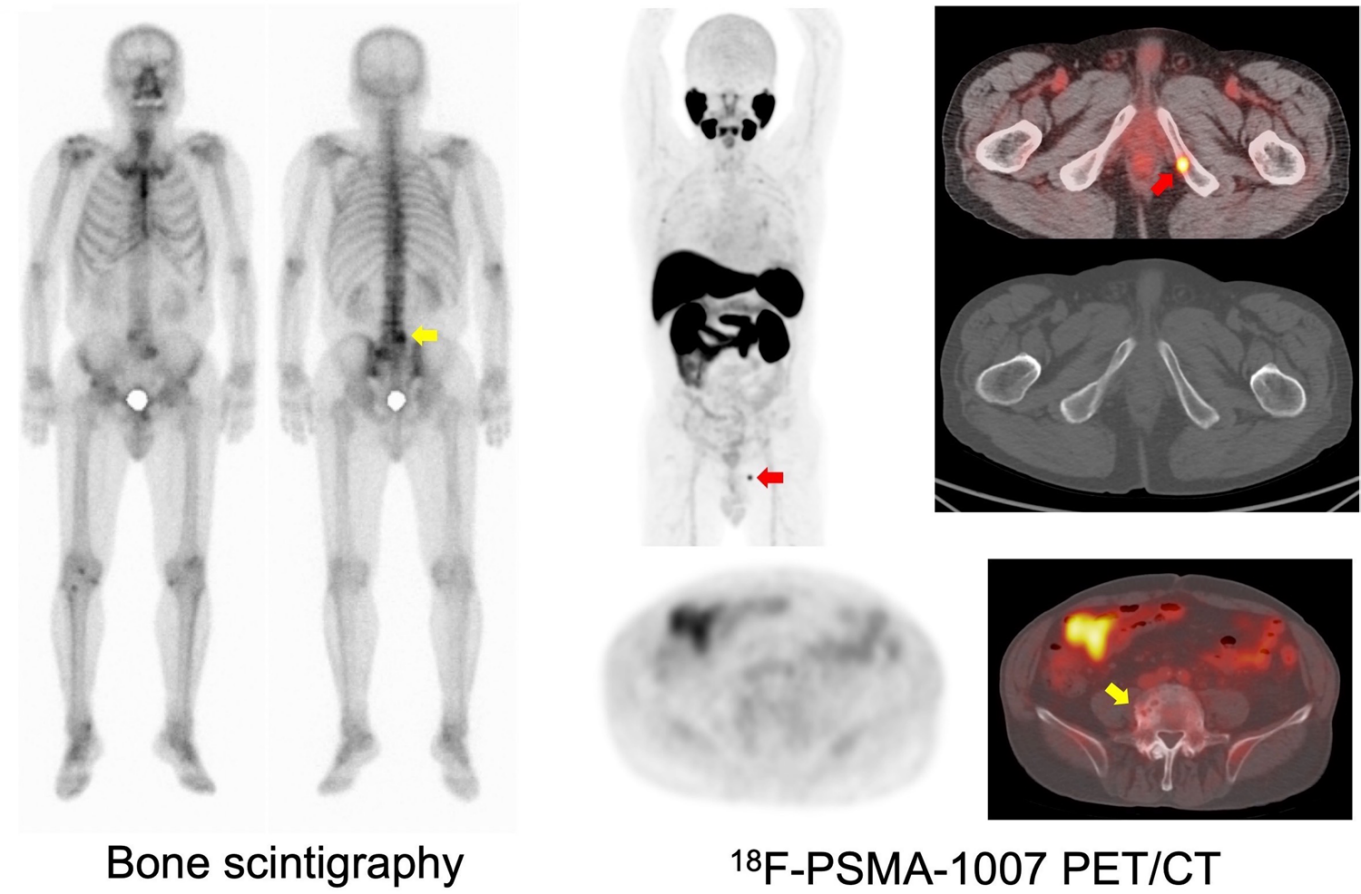
BCR patient after radical prostatectomy (PSA at PET: 1.7 ng/mL). Bone scintigraphy shows abnormal uptakes (yellow arrow) on the lower lumbar spine, which is considered to be a degenerative change. PSMA-PET shows focal uptake (red arrows) on the left ischium; whereas, it shows no significant uptake on the lower lumbar spine

Abnormal uptake, which was suspected to be recurrence or metastasis, was detected in 92.9% (26/28) of the patients with BCR. The SUVmax was 8.4 ± 6.4 in local recurrence, 11.5 ± 11.8 in pelvic lymph nodes (LN), and 4.1 ± 1.6 in bone metastasis (Fig. 1). The detection rates were 66.7% in the PSA group-1 (0.1–0.5 ng/mL), 85.7% in the PSA group-2 (0.5–1.0 ng/mL), and 100% in the PSA group-3 (above 1.0 ng/mL) (Fig. 2a). Among the PET-positive BCR patients (*n* = 26), local recurrence was detected in 57.7% (15/26), pelvic LN in 42.3% (11/26), and bone metastasis in 15.4% (4/26) (Fig. 2b). Representative cases are shown in Fig. 3. In patient with BCR after ^125^I-seed implantation, PSMA-PET showed a local recurrence with focal uptakes which was confirmed by biopsy. As shown in Fig. 4, PSMA-PET detected bone lesions without sclerotic lesions on CT, indicating the early stage of bone metastasis. Follow-up CT (11 months after PSMA-PET) showed osteosclerotic lesion in the PSMA-positive lesion on the left ischium, compatible with bone metastasis.

The location of the detected recurrence and its impact on patient management in the patients with BCR are shown in Table 2. The treatment strategy was finalized based on the results of PSMA-PET in 78.6% of BCR patients.

**Table 2:** (a) Location of the detected recurrence on [^18^F]PSMA-1007 PET and its relation to the treatment history in BCR patients (*n* = 28). Radiation therapy included ^125^I-seed implantation therapy (*n* = 2). (b) Impact on patient management after [^18^F]PSMA-1007 PET in BCR patients

Treatment history	Number of patients	
	Local recurrence	Metastasis
Radiation therapy (RT)	8/9 (88.9%)	1/9 (11.1%)
Radical prostatectomy	5/11 (45.5%)	4/11 (36.4%)
Radical prostatectomy and salvage RT	1/8 (12.5%)	7/8 (87.5%)

Impact on management	Number of patients
The treatment strategy was properly decided from PSMA-PET	22 / 28 (78.6%)
The decision by PSMA-PET was correct in patients treated with RT when evaluated from PSA response	11/11 (100%)

## Discussion

**Fig. 5:**
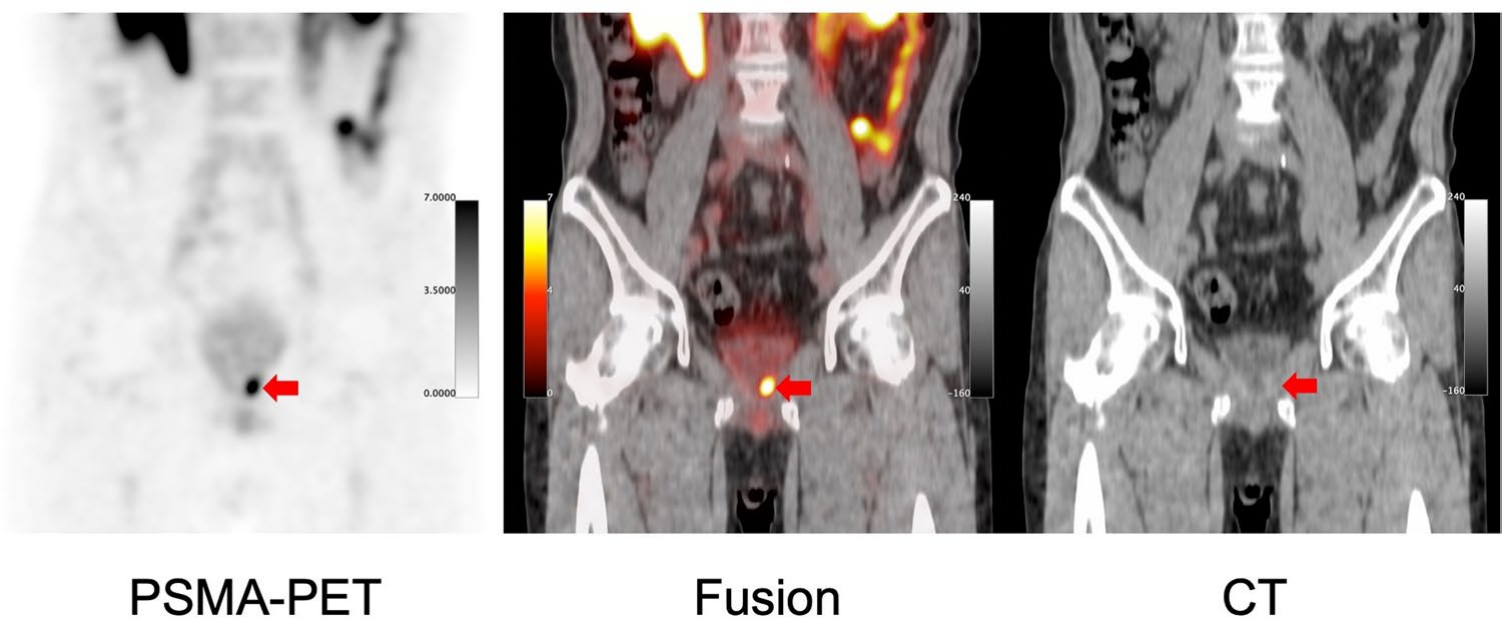
BCR patient after radical prostatectomy. Focal uptakes (red arrows) are detected at the edge of the bladder on [^18^F]PSMA-1007 PET (PSA at PET: 0.19 ng/mL). The patient is treated by radiation therapy targeting the PSMA-positive lesion and PSA values return to normal level (< 0.01 ng/mL)

In this study, [^18^F]PSMA-1007 PET had a high detection rate in patients with BCR. Since urine excretion is minimal in [^18^F]PSMA-1007 PET, it detects local recurrence more clearly than other PSMA-PET probes. In 53% (8/15) of BCR patients, who were suspected of local recurrence, focal uptake was detected adjacent to the bladder on [^18^F]PSMA-1007 PET. This suggested its significant advantage of less physiological urine excretion (Fig. 5). In addition, its high detection rate led to determining the proper treatment strategy, particularly local salvage radiation therapy, and boosting PSMA-positive lesions. These findings were consistent with those of previous reports [6, 11].

In BCR patients who had negative findings on conventional CT or BS, the PSMA-positive lesions were mainly smaller lymph nodes or nodules undetectable by CT. In comparison to BS, PSMA-PET was more effective in detecting small lesions with minimal osteosclerosis or excluded degenerative change although BS is sufficiently sensitive to detect small bone metastasis in most of the recurrent cases (data not shown). However, caution is advised when interpreting bone uptake of [^18^F]PSMA-1007, especially in the ribs, considering false-positive findings [12]. In addition, PSMA is not specific to prostate cancer. Some cancer lesions showed high PSMA uptake, mimicking metastasis of prostate cancer on PSMA-PET [13].

## Conclusions

[^18^F]PSMA-1007 PET showed a high detection rate in recurrent and metastatic lesions. In patients with BCR, its high detection led to proper treatment strategies, such as salvage radiation therapy or surgical removal of recurrent lymph nodes.
